# Non-Invasive Assessment of Treatment Response in Actinic Keratosis: A Clinically Oriented Multimodal Review

**DOI:** 10.3390/cancers18040708

**Published:** 2026-02-22

**Authors:** Gianluca Pistore, Luca Ambrosio, Antonio Di Guardo, Anna Rita Panebianco, Giovanni Di Lella, Claudio Conforti, Giovanni Pellacani, Francesco Moro, Paolo Marchetti, Damiano Abeni, Luca Fania, Francesco Ricci

**Affiliations:** 1 Clinical Epidemiology Unit, Istituto Dermopatico dell’Immacolata-IRCCS, 00167 Rome, Italy; gianluca@gianlucapistore.com (G.P.); d.abeni@idi.it (D.A.); 2 Dermatology Clinic, Department of Clinical Internal, Anesthesiological and Cardiovascular Sciences, La Sapienza University, 00185 Rome, Italy; giovanni.pellacani@uniroma1.it; 3 Skin Cancer Center, Istituto Dermopatico dell’Immacolata-IRCCS, 00167 Rome, Italy; l.ambrosio@idi.it (L.A.); a.diguardo@idi.it (A.D.G.); g.dilella@idi.it (G.D.L.); c.conforti@idi.it (C.C.); 4 Medical Direction, Istituto Dermopatico dell’Immacolata-IRCCS, 00167 Rome, Italy; a.panebianco@idi.it; 5 Department of Life Science, Health, and Health Professions, Link University of Rome, 00165 Rome, Italy; f.ricci@idi.it; 6 Dermatology Unit, Istituto Dermopatico dell’Immacolata-IRCCS, 00167 Rome, Italy; f.moro@idi.it; 7 Department of Oncology, Istituto Dermopatico dell’Immacolata-IRCCS, 00167 Rome, Italy; p.marchetti@idi.it; 8 Istituto Dermopatico dell’Immacolata di Roma, Via Monti di Creta 104, 00167 Roma, Italy; 9 Melanoma Unit, Istituto Dermopatico dell’Immacolata-IRCCS, 00167 Rome, Italy

**Keywords:** actinic keratosis, field cancerization, non-invasive imaging, treatment response, reflectance confocal microscopy, optical coherence tomography

## Abstract

Background: In actinic keratosis (AK), clinical clearance after field-directed therapies does not necessarily correspond to histological resolution, resulting in subclinical persistence and risk of recurrence. Objective: To provide a practical, up-to-date framework for non-invasive monitoring of treatment response in AK, integrating clinical assessment and dermoscopy with high-resolution imaging techniques, reflectance confocal microscopy (RCM), line-field confocal optical coherence tomography (LC-OCT), and high-frequency ultrasound (HFUS), and to discuss emerging optical biomarkers based on Raman spectroscopy. Results: For each modality, we summarize pre- and post-treatment imaging patterns, proposed response criteria, recommended follow-up timing, and correlations with clinical outcomes (including clearance and AKASI) and, when available, histological findings. The available evidence is derived from a limited number of observational studies, predominantly involving RCM and LC-OCT, whereas data on HFUS and Raman spectroscopy remain comparatively scarce. RCM and LC-OCT allow in vivo assessment of epidermal architectural normalization and reduction of intraepidermal keratinocyte atypia. HFUS captures quantitative trajectories of superficial dermal remodeling, including changes in the subepidermal low-echogenic band (SLEB) and dermal echogenicity after photodynamic therapy and other field treatments. Dermoscopy remains the first-line tool for routine follow-up but may fail to detect minimal subclinical persistence. Finally, we discuss the potential role of in vivo Raman spectroscopy for dynamic molecular endpoints and its possible integration with artificial intelligence–based analytical approaches. Conclusions: A standardized multimodal follow-up strategy improves the accuracy of treatment-response assessment compared with clinical evaluation alone. We propose a technique-specific checklist of minimal response criteria and a pragmatic temporal assessment scheme, and outline a research roadmap to support validation and clinical implementation of non-invasive imaging-guided monitoring in actinic keratosis.

## Simple Summary

Actinic keratoses are common skin lesions caused by long-term sun damage and may progress to skin cancer. After field-directed treatments, such as photodynamic therapy or topical drugs, lesions often appear clinically healed, but microscopic disease may persist and lead to recurrence. This review explains why clinical examination and dermoscopy alone may not be sufficient to assess treatment success, and discusses how non-invasive imaging techniques can improve follow-up. We summarize how advanced tools, including reflectance confocal microscopy, line-field optical coherence tomography, and high-frequency ultrasound can detect subtle residual disease and tissue recovery that are not visible to the naked eye. We also describe emerging optical approaches that analyze tissue chemistry. By integrating these methods in a multimodal strategy, clinicians may better evaluate treatment response, reduce unnecessary biopsies, and improve long-term management of sun-damaged skin.

## 1. Introduction

Actinic keratoses (AKs) represent the clinical manifestation of cutaneous field cancerization and are biologically positioned along a continuum that may culminate in squamous cell carcinoma (SCC). Their natural history is characterized by marked histopathological heterogeneity, variable clinical presentation (grades I–III), and the frequent presence of subclinical lesions within chronically photodamaged skin, all of which complicate the assessment of true disease burden and progression risk [1].

Field-directed therapies, including photodynamic therapy (PDT), 5-fluorouracil, imiquimod, tirbanibulin, and diclofenac, are specifically designed to target both clinically visible lesions and the surrounding subclinical disease within the cancerized field [2]. In routine clinical practice, treatment response is primarily evaluated through clinical inspection and dermoscopy. However, growing evidence indicates that clinical clearance does not necessarily reflect complete biological resolution. Histopathological persistence has been reported in up to 40–60% of lesions that appear clinically resolved, highlighting a substantial discrepancy between visible improvement and residual intraepidermal atypia.

Although dermoscopy is widely accessible, rapid, and highly effective in the diagnostic differentiation between AK and SCC, its performance in treatment monitoring is more limited. Morphological criteria of response may be subtle, transient, or nonspecific, particularly in thin AKs or following field therapies, reducing its sensitivity for detecting minimal residual disease [3]. As a result, dermoscopy alone may underestimate persistent subclinical alterations within the treated field.

This clinicopathological mismatch underscores the need for non-invasive imaging tools capable of documenting epidermal architectural normalization, reduction of keratinocyte atypia, and quantitative changes in the superficial dermis. High-resolution imaging techniques such as reflectance confocal microscopy (RCM), line-field confocal optical coherence tomography (LC-OCT), and high-frequency ultrasound (HFUS), together with emerging optical approaches such as in vivo Raman spectroscopy, have been proposed to address this unmet need by enabling a more comprehensive evaluation of treatment response, residual subclinical disease, and potential recurrence risk. Importantly, these imaging-derived parameters may complement established clinical trial endpoints, such as complete clearance rates, the Actinic Keratosis Area and Severity Index (AKASI), and recurrence, by providing a more sensitive, biologically grounded assessment of response beyond macroscopic appearance.

RCM and LC-OCT provide complementary cellular-to-architectural information, allowing in vivo visualization of epidermal disorganization, keratinocyte atypia, and their dynamic modification following therapy [4,5]. HFUS, while lacking cellular resolution, captures quantitative dermal changes, such as variations in subepidermal low-echogenic band (SLEB) thickness and dermal echogenicity, that reflect the evolution of the photodamaged field after treatment [6]. More recently, Raman spectroscopy has emerged as a potential tool for probing tissue biochemical composition, offering the possibility of identifying optical biomarkers associated with keratinization, dysplasia, and treatment-induced molecular changes [7].

In this narrative review, we propose a clinically oriented multimodal framework for monitoring treatment response in AK. Specifically, we aim to: (i) clarify the strengths and limitations of clinical assessment and dermoscopy in post-treatment follow-up; (ii) synthesize current evidence on response criteria derived from RCM, LC-OCT, and HFUS, with practical considerations regarding timing and standardization; and (iii) discuss the emerging, yet still exploratory, role of Raman spectroscopy as a potential surrogate molecular endpoint. Finally, we outline a pragmatic follow-up algorithm and a research roadmap to support future validation of non-invasive response criteria against clinically meaningful outcomes.

## 2. Materials and Methods

This work was designed as an evidence-informed narrative review aimed at summarizing the current state of the art and the clinical applicability of non-invasive imaging techniques for monitoring treatment response in actinic keratosis (AK) treated with field-directed therapies.

### 2.1. Literature Search Strategy

A structured literature search was conducted in PubMed and Scopus, covering publications from January 2010 to December 2025. For the PubMed search, official Medical Subject Headings (MeSH) were used when available, including “Keratosis, Actinic”, “Microscopy, Confocal”, “Tomography, Optical Coherence”, “Ultrasonography”, and “Dermoscopy”. These terms were combined with free-text keywords referring to imaging techniques without a dedicated MeSH term, such as “LC-OCT”, “high-frequency ultrasound”, “Raman spectroscopy”, “reflectance confocal”, “RCM”, “OCT”, and “HFUS”.

Keywords related to treatment response assessment were searched as free text and included “treatment response”, “monitoring”, “follow-up”, “post-treatment”, and “therapy”.

The complete PubMed search string was as follows: (“Keratosis, Actinic” [MeSH Terms] OR “actinic keratosis” [Title/Abstract] OR AK [Title/Abstract]) AND (“Microscopy, Confocal” [MeSH Terms] OR “reflectance confocal” [Title/Abstract] OR RCM [Title/Abstract] OR “Tomography, Optical Coherence” [MeSH Terms] OR OCT [Title/Abstract] OR “LC-OCT” [Title/Abstract] OR “Ultrasonography” [MeSH Terms] OR “high frequency ultrasound” [Title/Abstract] OR HFUS [Title/Abstract] OR “Dermoscopy” [MeSH Terms] OR dermoscopy [Title/Abstract] OR “Raman spectroscopy” [Title/Abstract] OR Raman [Title/Abstract]) AND (“treatment response” [Title/Abstract] OR monitoring [Title/Abstract] OR “follow up” [Title/Abstract] OR therapy [Title/Abstract] OR “post treatment” [Title/Abstract]).

The search was complemented by manual screening of the reference lists of selected articles. When appropriate, expert input from clinicians with experience in the management of AK and field cancerization was used to contextualize imaging findings. This expert input was limited to interpretative and contextual purposes and did not influence study selection or data extraction.

### 2.2. Eligibility Criteria and Study Selection

In PubMed, article types relevant to primary research were considered, including Clinical Study, Observational Study, Evaluation Study, Comparative Study, and Clinical Trial. Case reports and systematic reviews were excluded, in line with the predefined eligibility criteria.

Systematic reviews were excluded because the primary objective of the present work was to analyze original imaging-based response criteria, follow-up timing, and modality-specific performance rather than to summarize aggregated outcomes across heterogeneous study designs.

Eligible studies included investigations conducted in adult patients with AK treated with field-directed therapies such as photodynamic therapy, 5-fluorouracil, imiquimod, tirbanibulin, or diclofenac. To be included, studies were required to report treatment response assessed using at least one of the following non-invasive techniques: dermoscopy, reflectance confocal microscopy (RCM), line-field confocal optical coherence tomography (LC-OCT), high-frequency ultrasound (HFUS), or Raman spectroscopy, with evaluations performed both before and after treatment.

Studies were excluded if they consisted solely of isolated case reports, addressed diagnostic performance without post-treatment follow-up, were conducted exclusively on non-human skin, or lacked a full-text version available in English or Italian.

### 2.3. Data Extraction and Synthesis

Study selection was performed independently by two reviewers (F.R. and G.P.). For each included study, data were extracted on study design, type of field-directed therapy, number of patients or lesions evaluated, follow-up timing, and imaging-based criteria used to define treatment response or persistence of disease.

Given the narrative and non-systematic nature of this review, several intrinsic methodological limitations are acknowledged. Study inclusion, although based on predefined criteria, was influenced by the quality of reporting, full-text availability, and heterogeneity in terminology used to index imaging techniques, resulting in an unavoidable risk of selection bias. Furthermore, substantial variability in acquisition protocols, device-specific parameters, and definitions of response across studies introduced marked heterogeneity, precluding quantitative comparison of outcomes and rendering formal meta-analysis inappropriate. Publication bias is also likely, as studies reporting higher diagnostic performance or favorable results may be preferentially published.

Results were therefore synthesized using a descriptive and comparative approach, with particular emphasis on the ability of each imaging modality to detect disease resolution or persistent subclinical alterations, and on their potential role within a personalized follow-up strategy for field cancerization.

To enhance transparency and traceability of the study selection process, the phases of identification, screening, eligibility assessment, and inclusion were summarized using a PRISMA-adapted flow diagram, tailored to the requirements of a narrative review.

A limited number of key background references not retrieved through the structured search were manually added to provide contextual information on imaging techniques; these references were not included in the reviewed study pool.

## 3. Results

### 3.1. Study Selection and Evidence Overview

**Figure 1:**
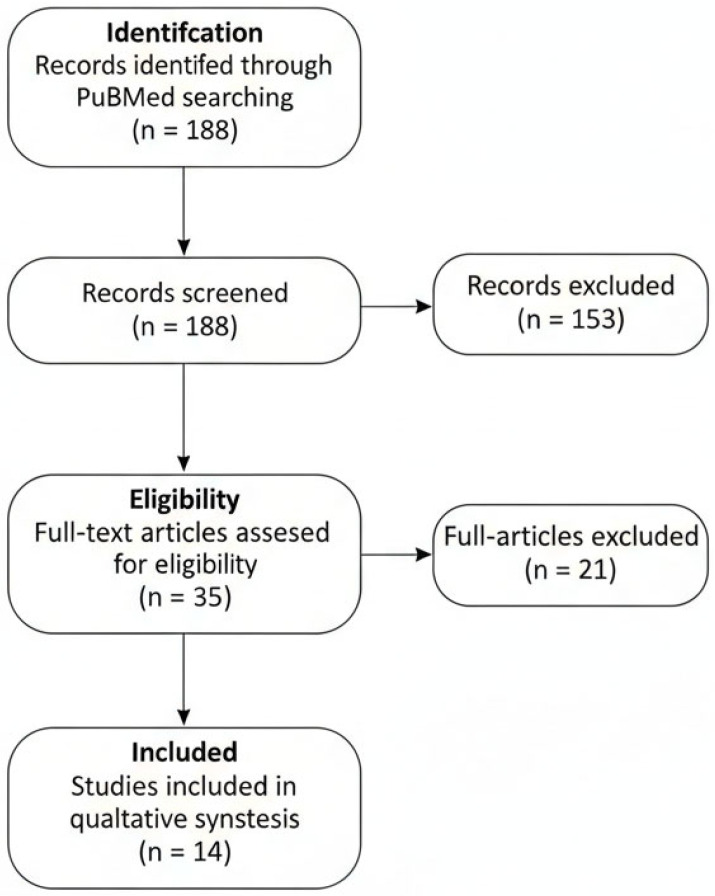
PRISMA-adapted flow diagram illustrating the identification, screening, eligibility assessment, and inclusion of studies in this narrative review. A total of 188 records were identified through database searching. After title and abstract screening, 35 full-text articles were assessed for eligibility. Fourteen studies met the predefined inclusion criteria and were included in the qualitative synthesis. The flowchart is adapted to reflect the methodology of an evidence-informed narrative review.

The literature search identified a total of 188 records. After screening for study type, language, and exclusion of preprints, 35 articles remained eligible for full-text assessment. Of these, 14 studies met the predefined inclusion criteria by reporting a pre- and post-treatment evaluation using at least one non-invasive imaging modality, and were therefore analyzed in detail (Figure 1). Most available evidence derives from studies based on RCM and LC-OCT, whereas data on HFUS and Raman spectroscopy remain quantitatively limited. A summary of included studies on non-invasive imaging for monitoring treatment response in actinic keratosis is included in Table 1.

### 3.2. Operational Definitions of Imaging-Based Response Criteria

For clarity, the following terms are used throughout this review with modality-specific meaning. Biological response refers to imaging evidence of epidermal architectural normalization together with the absence of residual keratinocyte atypia, when applicable, supported by parameters that have been shown to correlate with histological clearance, primarily with reflectance confocal microscopy (RCM) and line-field confocal optical coherence tomography (LC-OCT). Architectural normalization indicates restoration of a regular epidermal structure, such as a regular honeycomb pattern at RCM and a normalized epidermal thickness and dermo-epidermal junction contour at LC-OCT. Subclinical persistence denotes the persistence of imaging-detected cytologic atypia or structural abnormalities in the absence of corresponding clinical or dermoscopic signs of active disease.

### 3.3. Clinical Assessment and Dermoscopy: Strengths and Limitations

Clinical evaluation remains the first-line approach for follow-up after field-directed therapies in AK. In routine practice and in most registration trials, treatment success is commonly defined by the macroscopic regression of erythema, scaling, and surface roughness. However, several studies have demonstrated that clinical healing does not necessarily correspond to histological clearance, with persistence of subclinical disease reported within the cancerized field [21].

Dermoscopy provides a second level of resolution, allowing visualization of structural disorganization beyond naked-eye inspection. Its widespread availability, rapid execution, and high reproducibility make it particularly suitable for systematic monitoring of both individual lesions and the surrounding field. High diagnostic accuracy has been reported for AK, with sensitivities up to 98.7% and specificities around 95% [22].

From a diagnostic standpoint, dermoscopy improves discrimination between AK, pigmented AK (PAK), and SCC in situ through well-established criteria. Non-pigmented AKs typically display adherent white scales, an erythematous pseudonetwork, perifollicular keratinization, and the characteristic “strawberry pattern” whereas PAKs more often show gray–brown granules and globules, annular–granular structures, and an irregular pigmented pseudonetwork. SCC (both in situ and invasive) more frequently exhibits prominent hyperkeratosis, structureless white areas, irregular linear or glomerular vessels, and a higher degree of architectural disorganization.

Dermoscopy-associated signs of response include reduction or disappearance of keratin scales, attenuation of the erythematous pseudonetwork or strawberry pattern, resolution of superficial erosions or micro-ulcerations, and reduction of disorganized vascular structures. Owing to its immediacy, dermoscopy enables sequential assessment of post-treatment changes and may facilitate early detection of incomplete responses or progression toward SCC.

Nevertheless, dermoscopy may fail to detect minimal residual intraepidermal atypia or heterogeneous keratinocyte proliferation, particularly in initially thin lesions or after partially effective field therapies. As such, dermoscopy represents an excellent macroscopic and semiological tool, but is insufficient to definitively document biological resolution of disease [23,24,25].

### 3.4. Reflectance Confocal Microscopy (RCM)

RCM enables in vivo visualization of epidermal microarchitecture and the papillary dermis with near-cellular resolution. In AK, multiple studies have shown that RCM can capture dynamic patterns of response to field-directed therapies.

Before treatment, typical RCM findings include mosaic hyperkeratosis and parakeratosis, disruption of the epidermal “honeycomb” pattern, keratinocyte atypia of variable degree and distribution (dyskeratosis), blurring or loss of dermo-epidermal junction (DEJ) definition, increased superficial vascularization, and scattered inflammatory cells at the DEJ [26,27,28]. Following effective treatment, RCM commonly demonstrates restoration of a regular honeycomb pattern, increased uniformity of keratinocyte size and shape, reduction or disappearance of atypical keratinocytes, decreased inflammatory infiltrate, and normalization of superficial vascular structures [4,11,16,24,29,30,31,32].

Among RCM response criteria, it is possible to distinguish well-validated features, supported by histopathological correlation and prognostic relevance, from promising but less consolidated findings. Validated criteria include normalization of the honeycomb pattern, significant reduction of keratinocyte atypia, reconstitution of the DEJ, and disappearance of intraepidermal inflammatory cells. These parameters have shown reproducible association with histological clearance and reduced recurrence risk.

Other findings, such as changes in superficial vascular architecture, inflammatory dynamics, or subtle intraepidermal microvariants, remain less consistently validated and require further investigation to establish their prognostic value.

Importantly, architectural normalization observed by RCM has been associated with a lower risk of recurrence [14,31] supporting a prognostic, not merely diagnostic, role. In this context, RCM provides a functional surrogate of histological healing without the need for tissue sampling. Reported diagnostic performance of RCM in AK ranges from 79–100% sensitivity and 78–100% specificity [33].

### 3.5. Line-Field Confocal Optical Coherence Tomography (LC-OCT)

LC-OCT combines features of confocal microscopy and conventional OCT, enabling three-dimensional visualization of epithelial structures with near-micrometric resolution, albeit with lower cellular detail than RCM. It is particularly suited for quantifying epidermal structural changes before, during, and after treatment.

In pre-treatment AK, LC-OCT typically reveals increased thickness of the stratum corneum and epidermis (hyperkeratosis with parakeratosis and acanthosis), an irregular DEJ profile, hyperreflective inflammatory cells at the DEJ, and marked keratinocyte heterogeneity in intermediate layers (dyskeratosis), consistent with histopathological correlations. After treatment, progressive reduction of epidermal thickness, sharper and more continuous DEJ contours, increased keratinocyte uniformity, and decreased intraepidermal and junctional inflammatory cells have been documented in follow-up studies, particularly after tirbanibulin and other field therapies [34,35].

From an operational perspective, it is essential to distinguish parameters directly available on the device from those requiring dedicated post-processing. In routine practice, LC-OCT currently allows direct measurement of basic structural features, most notably epidermal thickness derived from vertical scans. More advanced quantitative parameters, such as automated assessment of keratinocyte atypia or numerical estimation of DEJ regularity, are not yet available in real time and require post-processing through manufacturer software or dedicated algorithms.

Within this context, the PRO-score, a composite AI-derived index combining multiple LC-OCT morphological features into a single atypia score, represents a promising but still investigational tool, currently limited to post-processing workflows and not integrated into standard clinical practice [5,36,37].

Recent studies on tirbanibulin-treated AK have reported early morphological normalization on LC-OCT (7–14 days), preceding visible clinical improvement typically observed at 4–8 weeks. Reported sensitivity and specificity for detecting AK-related structural abnormalities range from 85–90% and 82–89%, respectively [34]. These findings suggest that LC-OCT may identify subclinical residual disease and response dynamics earlier than dermoscopy and, in some settings, earlier than RCM, making it particularly valuable for short-course therapies.

### 3.6. High-Frequency Ultrasound (HFUS)

High-frequency ultrasound (HFUS) is a non-invasive imaging modality capable of exploring the superficial dermis at frequencies of approximately 20–50 MHz. Although it lacks cellular resolution, HFUS is particularly useful for assessing global changes within the cancerized field, especially after therapies such as PDT or topical 5-fluorouracil.

Pre-treatment HFUS findings typically include thickening of the subepidermal low-echogenic band (SLEB), a marker of chronic UV damage and collagen alteration, together with reduced echogenicity of the superficial dermis and, in some cases, non-quantifiable epidermal thickening, as reported in trials using HFUS as an outcome measure [19].

Following effective field therapy, HFUS commonly demonstrates a progressive reduction in SLEB thickness, often detectable within 4–8 weeks, accompanied by increased dermal echogenicity, interpreted as an indicator of improved tissue quality and dermal matrix reorganization. These changes have been shown to correlate with clinical and dermoscopic improvement and may remain stable over time.

Despite its value in assessing field cancerization, HFUS has important limitations. Epidermal resolution is insufficient to identify residual keratinocyte atypia or confirm complete lesion clearance, rendering cellular assessment of treatment response impossible. The technique is of limited utility in thin or minimally hyperkeratotic AK, where ultrasonographic parameters may be subtle or undetectable. Moreover, the lack of standardized response criteria and the strong influence of baseline photodamage introduce substantial heterogeneity across studies.

Overall, HFUS is most informative in patients with marked chronic actinic damage, where improvement in global skin quality represents a relevant therapeutic goal alongside lesion clearance. Its role remains complementary to higher-resolution epidermal techniques such as RCM and LC-OCT and requires further standardization to contribute more robustly to response assessment.

### 3.7. Raman Spectroscopy: Emerging Optical Biomarkers

Raman spectroscopy enables acquisition of an in vivo molecular fingerprint of the skin, providing information on tissue biochemical composition. In the context of AK follow-up, this technique may detect changes related to keratinization, dysplasia, and inflammatory processes, potentially allowing monitoring of treatment response through dynamic spectral modifications.

Initial studies exploring Raman spectroscopy in post-treatment monitoring have reported preliminary response-associated patterns. In a case report [7] and a study [38], spectral analysis following PDT, tirbanibulin, or 5-fluorouracil revealed changes in bands attributed to elastin (≈1680–1690 cm^−1^) and tyrosine/keratin (≈1610–1620 cm^−1^). Progressive reduction of these signals, combined with multivariate models such as principal component analysis (PCA) or partial least squares (PLS), was preliminarily associated with biochemical regression of the lesion, in some cases preceding clinically visible changes.

While Raman spectroscopy represents a promising approach for defining a non-invasive biological endpoint complementary to morphological imaging, current evidence remains exploratory, limited to small cohorts, and lacks validated, reproducible criteria. Further controlled studies are required before routine clinical implementation can be considered.

## 4. Discussion

Assessing treatment response in actinic keratosis (AK) after field-directed therapies remains a central clinical challenge in the management of field cancerization. In both routine practice and clinical trials, clinical clearance is still the most frequently adopted endpoint; however, a substantial body of evidence indicates that visible normalization does not necessarily correspond to true biological or histological resolution. A study [39] reported histological persistence in up to 60% of clinically “cleared” AK, highlighting a clinically relevant mismatch between macroscopic outcomes and the underlying intraepidermal disease status. This dissociation plausibly contributes to the variability in recurrence rates reported across field treatments such as photodynamic therapy (PDT), 5-fluorouracil, imiquimod, and tirbanibulin, where outcome estimates depend heavily on the definition of response and on the depth and sensitivity of post-treatment assessment.

Within this framework, dermoscopy remains an indispensable first-line tool. Its diagnostic performance at baseline is high (sensitivity up to 98.7% and specificity approximately 95%) [22], and it is uniquely positioned for wide implementation due to accessibility, speed, and reproducibility. Dermoscopy also supports structured assessment of lesion morphology and facilitates early recognition of features suspicious for progression toward squamous cell carcinoma (SCC). Nonetheless, its role in treatment-response monitoring is intrinsically constrained by the limited ability to detect minimal residual intraepidermal atypia or heterogeneous subclinical persistence within the treated field. In keeping with this limitation, Peppelman et al. reported histological persistence in approximately 40% of lesions no longer visible on dermoscopic examination. These observations support the concept that dermoscopy, while effective as a clinical decision tool, is not sufficient as a stand-alone surrogate of biological clearance when the clinical question is residual subclinical disease after field therapy. In Table 2, we summarize the sensitivity, specificity, and capability of non-invasive techniques in detecting subclinical AK after field therapy.

High-resolution, non-invasive imaging techniques address this gap by providing objective or semi-objective criteria closer to histopathology, while maintaining a non-invasive workflow. Reflectance confocal microscopy (RCM) is particularly relevant in this setting because it offers near-cellular visualization of epidermal architecture and superficial dermis and can document therapy-induced changes in a manner that is interpretable against known histologic correlates. Reported diagnostic performance for AK spans a sensitivity of 79–100% and specificity of 78–100% [34]. Beyond diagnosis, follow-up studies indicate that RCM detects persistent subclinical alterations in a clinically meaningful proportion of cases judged as cleared by clinical inspection, with higher concordance with histopathology than dermoscopy. Pellacani et al. observed that RCM can identify persistence in approximately 30–50% of clinically resolved cases. Importantly, the restoration of a regular honeycomb pattern and reduction of keratinocyte atypia have been repeatedly associated with histological clearance [14,31], supporting the use of RCM-derived architectural normalization as a pragmatic surrogate endpoint for “biological response.” These data position RCM as a technique capable of moving response assessment beyond macroscopic appearance toward a microstructural definition that may better align with recurrence risk.

Line-field confocal optical coherence tomography (LC-OCT) occupies a complementary position. By combining high axial resolution with the ability to interrogate tissue in three dimensions, LC-OCT enables serial quantification of epidermal and junctional morphology while retaining a clinically feasible acquisition process. In clinical studies, LC-OCT has shown sensitivity around 85–90% and specificity approximately 82–89% for AK-related abnormalities [34]. A consistent finding across follow-up series is that LC-OCT may reveal structural normalization early after therapy initiation, in some reports within the first 1–2 weeks, potentially preceding the timepoint at which clinical normalization becomes apparent. This characteristic is particularly relevant in short-course regimens, where early discrimination between expected inflammatory remodeling and incomplete response may influence follow-up strategy. Also a report [35] further supports the concept that dynamic OCT-based assessment can capture early reduction of epithelial alterations during tirbanibulin treatment. At the same time, it remains essential to distinguish what is currently achievable in routine LC-OCT workflows from what is only possible with advanced post-processing. Although basic structural measurements such as epidermal thickness can be derived directly from standard acquisitions, composite or automated indices of atypia (including AI-derived scores such as the PRO-score) are not uniformly available in real time and remain in validation phases in many settings. As such, LC-OCT can be framed as a high-resolution structural follow-up tool with strong potential for standardization, while avoiding overstatement regarding automated quantification until broader validation and implementation are available.

High-frequency ultrasound (HFUS) provides a different, complementary layer of information by focusing on dermal changes that reflect chronic photodamage and field remodeling rather than keratinocyte-level atypia. Several studies [35] have described reductions of the subepidermal low-echogenic band (SLEB) on the order of 20–25% and increases in dermal echogenicity after PDT, correlating with improvement in the photo-damaged dermal matrix. Although formal sensitivity and specificity values for detecting subclinical residual AK are generally not available for HFUS, its contribution lies in quantifying field-level dermal recovery and offering a potential imaging correlate of “field improvement” beyond lesion clearance. This may be clinically meaningful in patients with extensive actinic damage, where therapeutic goals include not only clearance of visible AK but also reduction in the propensity to develop new lesions. Nevertheless, HFUS should be considered complementary rather than confirmatory for lesion-level clearance, because epidermal resolution is insufficient to directly evaluate residual intraepidermal atypia.

Raman spectroscopy should currently be regarded as a translational research tool aimed at identifying molecular response signatures rather than as a near-term clinical monitoring modality. Its primary relevance lies in biomarker discovery and potential surrogate endpoint development within controlled clinical trials. Preliminary reports [7,38] suggest that dynamic changes in spectral bands related to keratin, elastin, and inflammatory pathways may accompany, and in some instances precede, clinically apparent response. While this is conceptually attractive as a potential molecular surrogate endpoint, the evidence base is currently early-stage, often limited by small sample sizes, heterogeneous acquisition protocols, and limited reproducibility across devices and analytical pipelines. Accordingly, Raman should be positioned as an investigational modality with potential utility in research settings and trial endpoints, rather than as a technique ready for routine response assessment.

Taken together, these modalities should not be framed as interchangeable alternatives, but as tools with distinct strengths that can be integrated into a rational follow-up strategy. Clinical evaluation and dermoscopy remain the foundation for screening, baseline characterization, and routine follow-up. RCM and LC-OCT can be deployed when the clinical question is whether residual subclinical disease persists despite apparent clinical response, or when a higher-confidence, microstructural definition of response is required (e.g., high-risk sites, recurrent disease, immunosuppression, or equivocal post-treatment findings). HFUS can add value by characterizing dermal field remodeling and monitoring broader photodamage-related changes. Raman spectroscopy, at present, is best reserved for exploratory applications and for the development of future biochemical endpoints within controlled studies.

The implementation of non-invasive imaging in AK follow-up is associated with limitations that should be explicitly acknowledged. From a technical standpoint, marked hyperkeratosis, severe photodamage, and alterations of the dermo-epidermal junction may impair image quality and reduce the ability to detect subtle residual disease. From an organizational and economic standpoint, cost, limited availability to specialized centers, acquisition time, and the requirement for advanced training constitute relevant barriers to broad implementation. A further limitation is the lack of standardized acquisition protocols and response definitions across devices and research groups, which limits comparability and hampers the development of shared decision pathways. Finally, the current evidence base is frequently derived from monocentric cohorts with modest sample sizes and relatively short follow-up, which constrains the strength and generalizability of conclusions regarding recurrence prediction and long-term outcomes.

Future progress in this field will depend on standardization and validation. Priority directions include: (i) consensus definitions of imaging-based response criteria for each modality, anchored to clinically meaningful outcomes and, where appropriate, histological correlation; (ii) harmonized acquisition and reporting standards to improve reproducibility across centers; (iii) prospective multicenter studies designed to evaluate whether imaging-guided follow-up reduces recurrence, enables earlier identification of incomplete response, or optimizes retreatment timing; and (iv) careful evaluation of AI-assisted metrics (including LC-OCT composite scores) with transparent methodology, external validation, and clinically interpretable thresholds. Within this roadmap, a multimodal approach has the potential to improve the precision of response assessment beyond clinical clearance alone, while maintaining feasibility and proportionality in real-world dermatology workflows.

**Figure 2:**
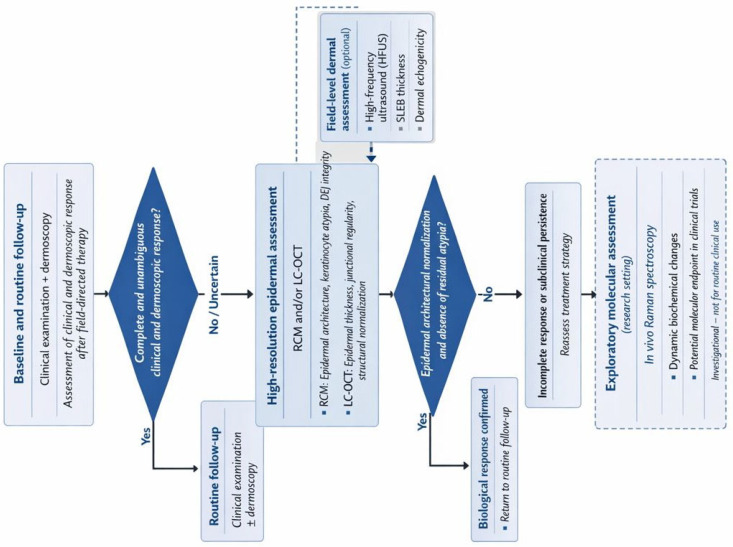
Conceptual multimodal follow-up algorithm for actinic keratosis (AK) after field-directed therapy. Clinical examination and dermoscopy represent the first-line assessment. In cases of incomplete or uncertain response, high-resolution epidermal imaging with reflectance confocal microscopy (RCM) and/or line-field confocal optical coherence tomography (LC-OCT) is proposed to evaluate architectural normalization and residual atypia. High-frequency ultrasound (HFUS) may complement follow-up by assessing dermal field remodeling, including the subepidermal low-echogenic band (SLEB), in selected patients. In vivo Raman spectroscopy is currently limited to exploratory research settings. This algorithm is intended as a pragmatic, non-validated framework primarily for lesion-level response assessment, with complementary field-level evaluation using HFUS. Its application is most appropriate in specialized or tertiary care settings.

From a practical standpoint, these findings suggest that non-invasive imaging should be applied in a risk-adapted and question-driven manner rather than uniformly in all patients. In everyday clinical practice, most AK can continue to be managed and followed with clinical examination and dermoscopy alone. Advanced imaging modalities become most relevant when clinical response is equivocal, when treatment is performed on high-risk anatomical sites, or when patients present with factors associated with increased recurrence risk, such as extensive field cancerization or immunosuppression. In this context, selective integration of RCM or LC-OCT may enhance confidence in response assessment and inform retreatment decisions, while HFUS may support longitudinal monitoring of field recovery. Such a proportional approach balances diagnostic accuracy with feasibility and resource utilization, facilitating translation of multimodal follow-up strategies into routine dermatologic care. Based on these considerations, we propose a clinically oriented, multimodal follow-up algorithm designed to support post-treatment decision-making in actinic keratosis. This framework is intended as a conceptual and pragmatic tool, rather than a validated guideline, and should be applied in a risk-adapted, question-driven manner (Figure 2). The proposed algorithm primarily addresses lesion-level response assessment following field-directed therapy, with HFUS contributing complementary information at the field level by characterizing dermal remodeling rather than confirming lesion clearance. Its implementation is most appropriate in tertiary referral settings or in selected high-risk scenarios where access to high-resolution imaging is available and clinically justified.

## 5. Conclusions

Follow-up of actinic keratosis should extend beyond clinical inspection and dermoscopy, which document visible regression but do not reliably confirm biological clearance. Non-invasive imaging techniques—reflectance confocal microscopy (RCM), line-field confocal optical coherence tomography (LC-OCT), and high-frequency ultrasound (HFUS)—provide complementary information by improving detection of epidermal normalization, subclinical persistence, and changes within the cancerized field. Raman spectroscopy, although still exploratory, further expands this framework by offering access to tissue-level biochemical changes.

An effective follow-up strategy is therefore multimodal, with each technique fulfilling a specific role: RCM for near-cellular assessment of keratinocyte atypia, LC-OCT for quantitative structural evaluation, HFUS for dermal field remodeling, and Raman spectroscopy as a potential molecular endpoint in research settings. Integrated use of these modalities can enhance response assessment and reduce the risk of unrecognized residual disease.

Future efforts should focus on standardizing response criteria, validating imaging endpoints in multicenter studies, and developing robust morphological and biochemical biomarkers, including those supported by artificial intelligence, to enable more reliable and sustainable monitoring of actinic keratosis in clinical practice.

## Figures and Tables

**Table 1:** Summary of included studies on non-invasive imaging for monitoring treatment response in actinic keratosis.

First Author, Year	Imaging Technique	Therapy	Size	Primary Outcome	Main Findings
**Banzhaf 2014** [8]	OCT	Imiquimod	20 patients (11 AK)	The ability of OCT to predict treatment outcome	OCT showed thinning of AKs indicating effect of treatment
**Fredman 2024** [9]	D-OCT	Daylight photodynamic therapy	38 patients (62 AK)	Quantitative vascular parameters on dynamic OCT to differentiate treatment-resistant vs. cleared AK.	D-OCT holds potential to identify treatment-resistant AKs.
**Themstrup 2014** [10]	OCT	MAL-PDT	18 patients (4 AK)	Describe the OCT morphology of in vivo NMSC lesions during PDT treatment and to investigate the use of OCT in evaluating the response of PDT treated NMSC lesions.	OCT is most suitable in the diagnosis and follow-up of NMSC treatment.
**Seyed Jafari 2016** [11]	RCM	DL-PDT	20 patients (40 AK)	Use a new RCM atypia scoring system to evaluate efficacy of DL-PDT	This study confirms that in vivo RCM technology might be an additional technique to monitor the efficacy of DL-PDT for AK
**Ulrich 2010** [12]	RCM	Imiquimod	11 patients	Applicability of RCM for noninvasive monitoring of AK	RCM allows noninvasive monitoring of treatment response in vivo and permits early detection of subclinical AK
**Qiao 2023** [13]	RCM	PDT + microneedling, fractional CO2 laser, and cryotherapy	129 patients	Complete response rate	RCM ismore sensitive than dermoscopy for identifying residual atypia. Shows superior cellular-level clearance with combination therapies, particularly cryotherapy + PDT.
**Ishioka 2018** [14]	RCM	5-fluorouracil	50 lesions	RCM accuracy, sensibility and specificity for actinic keratosis	RCM is a non-invasive method capable of monitoring actinic keratosis therapeutic response to 5-fluorouracil, presenting efficacy comparable to histological examination
**Pasquali 2018** [15]	RCM	IM and cryotherapy	26 patients	RCM-monitored treatment response	IM + Cryo shows fewer LSR; both sequences effective
**Ruini 2019** [16]	RCT and OCT	Ingenol mebutate	20 patients (120 lesions)	Evaluate the changes in the field cancerization undergoing treatment by combining RCM and OCT	Both OCT and RCM allow the morphological representation of field cancerization including subclinical lesions and provide complementary information.
**Richtig 2010** [17]	RCM	Shave biopsy	10 lesions	Applicability of RCM for the follow-up of AK after shave biopsy	RCM might be a useful tool in the follow-up of AK after shave biopsy
**Caccavale 2024** [18]	RCM	n-DL-PDT without curettage but preceded by application of keratolytics	39 patients	Evaluate efficacy, based on RCM assessments of the therapy	curettage is not necessary to obtain the full treatment effect of n-DL-PDT
**Arisi 2020** [19]	HFUS	MAL-PDT, IMB, DHA	90 patients	Evaluate dermoscopical and high-frequency ultrasound (HFUS) changes after therapies	MAL-PDT improves all HFUS features of chronic photodamages of the dermis of the skin underlying and surrounding the AK spots.
**Ishioka 2015** [14]	RCM	Fluorouracil	13 patients	Confocal microscopy enabled visualization of focal areas of atypical honeycomb pattern in the epidermis indicating therapeutic failure	RCM may be a tool for diagnosis and therapeutic control of actinic keratosis
**Scola 2012** [20]	OCT	PDT and CO(2)	20 patients	To compare PDT and carbon dioxide (CO(2)) LA in the management of multiple AK	AK features as assessed by OCT imaging were also significantly reduced by both procedures.

**Table 2:** Sensitivity, specificity and capability of non-invasive techniques in detecting subclinical AK after field therapy.

Method	Sensitivity (%)	Specificity (%)	Subclinical Residue Detection/Notes	Main Sources
**Dermoscopy**	85–98.7	82–95	High diagnostic accuracy at baseline. Limited ability to detect subclinical persistence (~40% histologic residue with normal dermoscopy).	Zalaudek 2013 [40]; Huerta-Brogeras 2015 [22]
**Reflectance Confocal Microscopy (RCM)**	79–100	78–100	Most accurate technique for detecting subclinical residue. Identifies 30–50% persistence in clinically “cleared” AK. Architectural normalization correlates with histologic clearance.	Malvehy 2016 [31]; Ishioka 2018 [14]
**Line-Field Optical Coherence Tomography (LC-OCT)**	85–90	82–89	Detects minimal residual abnormalities; early structural normalization (7–14 days). Particularly useful for short-course therapies.	Cinotti 2020 [41]; Cantisani 2024 [35]
**High-Frequency Ultrasound (HFUS)**	Not established for lesion-level subclinical detection	Not established for lesion-level subclinical detection	Does not detect cellular atypia; quantifies dermal recovery (SLEB reduction 20–25%; echogenicity ↑ 15–20%).	Zhu 2021 [42]; Korecka 2024 [6]; Dinnes 2017 [43]
**Raman spectroscopy**	80–92 *	78–90 *	Preliminary evidence only. Molecular bands (1610–1690 cm^−1^) predict early biochemical response. * Values from ML models. *	Zhu 2025 [7]; Zhao 2024 [38]

* Values derived from experimental machine-learning models; not directly comparable to clinical diagnostic sensitivity/specificity.

## Data Availability

No new data were created or analyzed in this study. Data sharing is not applicable to this article.
